# Effect of inoculation of *Burkholderia* sp. strain SJ98 on bacterial community dynamics and *para*-nitrophenol, 3-methyl-4-nitrophenol, and 2-chloro-4-nitrophenol degradation in soil

**DOI:** 10.1038/s41598-017-06436-0

**Published:** 2017-07-20

**Authors:** Jun Min, Bin Wang, Xiaoke Hu

**Affiliations:** 1 Key Laboratory of Coastal Biology and Bioresource Utilization, Yantai Institute of Coastal Zone Research, Chinese Academy of Sciences, Yantai, Shandong China; 2 University of Chinese Academy of Sciences, Beijing, China

## Abstract

*para-*Nitrophenol (PNP), 3-methyl-4-nitrophenol (3M4NP), and 2-chloro-4-nitrophenol (2C4NP) are highly toxic compounds that have caused serious environmental issues. We inoculated an artificially contaminated soil with *Burkholderia* sp. strain SJ98, which has the ability to degrade PNP, 3M4NP, and 2C4NP, and quantified bioremediation. There was accelerated degradation of all nitrophenols in inoculated treatments compared to the un-inoculated treatments. The indigenous bacteria were able to degrade PNP, but not 3M4NP or 2C4NP. Real-time PCR targeting the catabolic gene *pnpA* showed that levels of strain SJ98 remained stable over the incubation period. High-throughput sequencing revealed that both contamination and bioaugmentation influenced the bacterial community structure. Bioaugmentation seemed to protect *Kineosporia*, *Nitrososphaera*, and *Schlesneria* from nitrophenol inhibition, as well as led to a sharp increase in the abundance of *Nonomuraea*, *Kribbella*, and *Saccharopolyspora*. There was a significant increase in the relative abundances of *Thermasporomyces*, *Actinomadura*, and *Streptomyces* in both contaminated and bioaugmented treatments; this indicated that these bacteria are likely directly related to nitrophenol degradation. To our knowledge, this is the first report of the simultaneous removal of PNP, 3M4NP, and 2C4NP using bioaugmentation. This study provides valuable insights into the bioremediation of soils contaminated with nitrophenols.

## Introduction

Methyl-parathion (*O*,*O*-dimethyl *O*-*p*-nitrophenol phosphorothioate), fenitrothion (*O*,*O*-dimethyl *O*-*p*-nitro-*m*-tolyl phosphorothioate), and dicapthon [*O*,*O*-dimethyl *O*-(2-chloro-4-nitrophenyl) phosphorothioate] are representative organophosphorus pesticides that are used extensively to control a wide range of agricultural pests, especially in developing countries. In aerobic environments, methyl-parathion, fenitrothion, and dicapthon can be rapidly degraded (days to weeks^1–3^) to *para*-nitrophenol (PNP), 3-methyl-4-nitrophenol (3M4NP), and 2-chloro-4-nitrophenol (2C4NP); these compounds are persistent and have serious health impacts on humans and animals^4, 5^. These nitrophenol pollutants are also widely used in synthesizing drugs, dyes, herbicides, and fungicides, among others^4, 6^. Although they are less toxic than their parent chemicals, PNP, 3M4NP, and 2C4NP are highly water soluble and have been detected in agricultural soils, surface water, ground water, rain water, air, active sludge, and industrial effluents^4, 7–10^.

Bioaugmentation is the introduction of a specific microorganism or a microbial consortium to accelerate and/or enhance the removal of toxic compounds^11^, and is considered a feasible strategy in remediating contaminated soils^12, 13^. In recent decades, bioaugmentation has been used to treat soils contaminated with various recalcitrant aromatic pollutants, including PNP^14^, nitrobenzene^15^, 4-chloronitrobenzene^16^, 3-chloroaniline^17^, 4-chlorophenol^18^, and polycyclic aromatic hydrocarbons^19–21^. Bioaugmentation with a single microorganism has also been used to remediate soil contaminated with mixtures of PNP and other compounds^22, 23^. More recently, soils contaminated with PNP and two other isomers (i.e., *meta*-nitrophenol and *ortho*-nitrophenol) were remediated by augmentation with a microbial consortium^24^. The wide application of PNP, 3M4NP, and 2C4NP by the agricultural, pharmaceutical, and chemical industries over the past century has led to accumulation of these nitrophenols in soils. However, there has been no study investigating bioaugmentation for the simultaneous removal of PNP, 3M4NP, and 2C4NP by either a single microorganism or a microbial consortium.

Generally, a successful bioaugmentation needs a metabolically active inoculum of a microorganism or consortium able to survive in the complex *in-situ* environment^15, 21, 25, 26^. Real-time PCR-based quantification of catabolic gene copy numbers has been recently shown to be a more efficient and reliable technique than plate counting for assessing the fate of the inoculated strains during bioaugmentation^24, 27^. The basic premise of real-time PCR for detecting functional genes is that the catabolic pathways of the target pollutants in the inoculated strain are known at both the genetic and biochemical levels. Additionally, investigating the effects of exogenous microorganisms on the natural microbial community is important during bioaugmentation. It has been reported that an exogenous strain or strains can alter the structure and function of ecosystems^28^. Recently, high-throughput sequencing of 16S rRNA gene amplicons^19^ has replaced denaturing gradient gel electrophoresis for the characterization of soil bacterial community composition.

*Burkholderia* sp. strain SJ98 (originally classified as a *Ralstonia* sp.) is able to utilize PNP^29^, 3M4NP^30^, or 2C4NP^31^ as the sole source of carbon, nitrogen, and energy. Recently, we have elucidated the catabolic pathways and mechanisms of PNP, 3M4NP, and 2C4NP utilization in strain SJ98, and determined that the enzymes encoded by the *pnpABA1CDEF* cluster are responsible for the catabolism of these nitrophenols^32, 33^. Here, we report the successful bioaugmentation of an agricultural soil artificially contaminated with PNP, 3M4NP, and 2C4NP using strain SJ98. We monitored the effects of the bioaugmentation on bacterial abundance and community structure by high-throughput sequencing. This is the first report of the simultaneous removal of PNP and its methyl- and chloro-substituted derivatives using bioaugmentation, and provides valuable insight into the bioremediation of soils contaminated with nitrophenols.

## Results

### Degradation of nitrophenols and accumulation of nitrite

Six different concentrations [10, 20, 30, 50, 70, and 100 μg g^−1^ dry weight (dw)] of PNP, 3M4NP, and 2C4NP were initially added to both contamination and bioaugmentation treatments to determine the highest concentration that could be removed completely within a reasonable period of time. PNP, 3M4NP, and 2C4NP were completely degraded after a 30-day incubation period for concentrations less than or equal to 50 μg g^−1^ dw (Table S1). Therefore, for further experiments we used concentrations of 45 μg g^−1^ dw.

**Figure 1:**
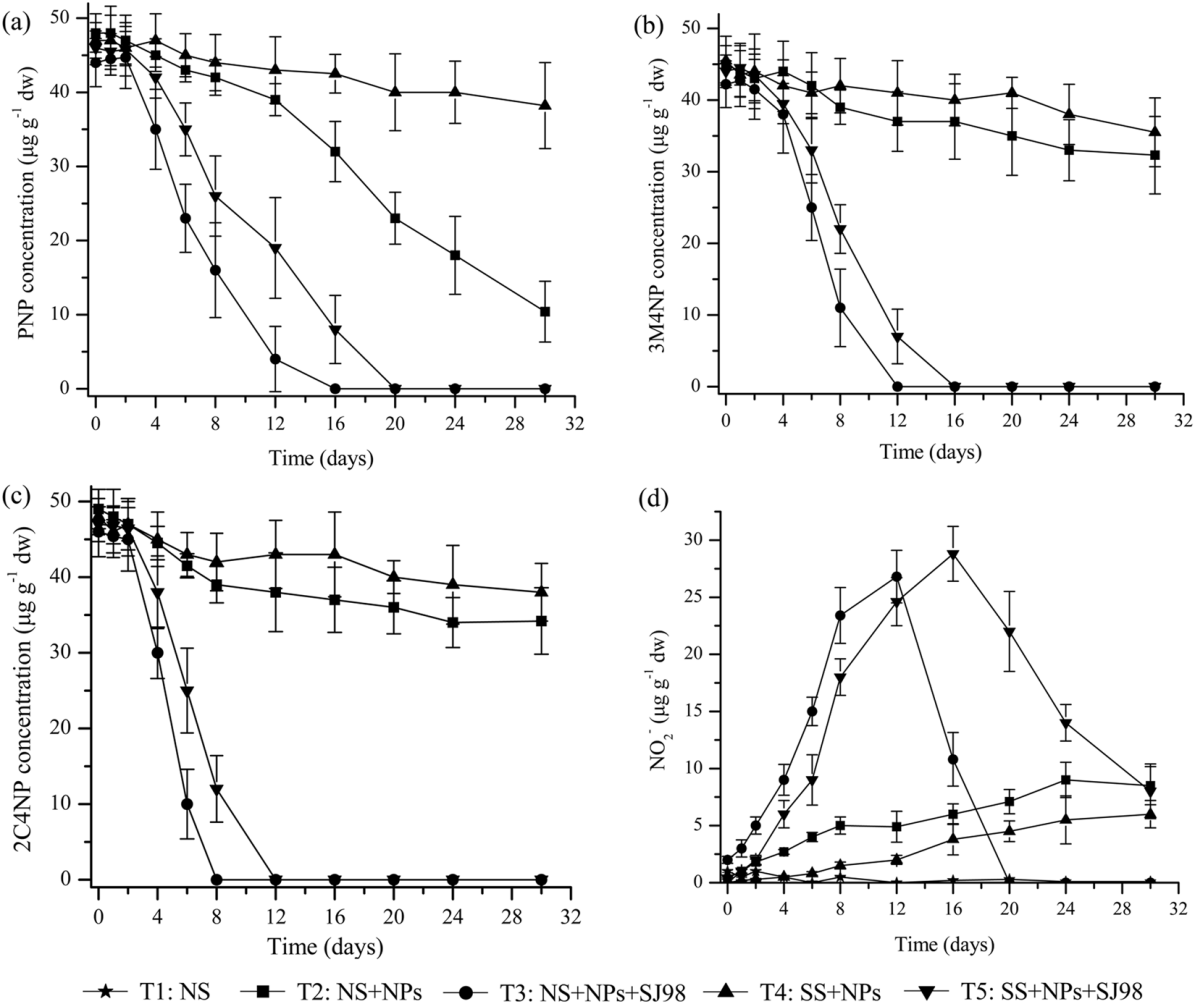
Concentration of nitrophenol pollutants and nitrite in soil microcosms: (**a**) *para*-nitrophenol (PNP); (**b**) 3-methyl-4-nitrophenol (3M4NP); (**c**) 2-chloro-4-nitrophenol (2C4NP), and (**d**) nitrite (NO_2_^−^). Values are means ± standard deviation (n = 3). NS: native soil, SS: sterilized soil, NPs: nitrophenols, and SJ98: *Burkholderia* sp. strain SJ98.

Subsequently, the capability of strain SJ98 to degrade a mixture of PNP, 3M4NP and 2C4NP was analyzed detailedly by setting up five different microcosm as following: (T1) native soil; (T2) native soil with nitrophenols added; (T3) native soil with nitrophenols added and with strain SJ98 inoculated; (T4) sterilized soil with nitrophenols added; (T5) sterilized soil with nitrophenols added and with strain SJ98 inoculated. Degradation of all three nitrophenols occurred more rapidly in both inoculated treatments (T3 and T5; Fig. 1) than in the un-inoculated treatments (T2 and T4). In the inoculated non-sterile soil (T3) and inoculated sterile soil (T5), PNP was completely removed by day 16 and 20, respectively. However, there was still PNP in the un-inoculated non-sterile soil (T2) and un-inoculated sterile soil (T4) after 30 days of incubation, with 22% (10.4 ± 4.1 μg g^−1^ dw) and 81% (38.2 ± 5.8 μg g^−1^ dw) of the initial PNP remained, respectively (Fig. 1a). 3M4NP was completely degraded in T3 and T5 (12 and 16 days into the incubation, respectively); however, in T2 and T4, 75% (32.3 ± 5.4 μg g^−1^ dw) and 82% (37.5 ± 4.8 μg g^−1^ dw), respectively, remained on day 30 (Fig. 1b). 2C4NP was removed completely from T3 and T5 by day 8 and 12, respectively; meanwhile, in T2 and T4, 70% (34.2 ± 4.4 μg g^−1^ dw) and 80% (38.5 ± 3.8 μg g^−1^ dw) remained on day 30, respectively (Fig. 1c).

Although PNP, 3M4NP, and 2C4NP were completely degraded in both bioaugmentation treatments (T3 and T5), different removal rates were observed for the three substrates. In T3, 2C4NP was completely removed by day 8; however, 34% (16.4 ± 6.4 μg g^−1^ dw) of the PNP and 26% (11.5 ± 5.4 μg g^−1^ dw) of the 3M4NP remained in the soil at this time. In T5, 2C4NP was completely removed by day 12; however, 42% (19.5 ± 6.8 μg g^−1^ dw) of the PNP and 17% (7.3 ± 5.8 μg g^−1^ dw) of the 3M4NP remained in the soil at this time. PNP was more rapidly degraded in the non-sterile treatment (T2) than in the sterile treatment (T4); meanwhile, 3M4NP and 2C4NP were not degraded in T2 or T4.

In addition to degradation of PNP, 3M4NP and 2C4NP, concomitant accumulation of nitrite (Fig. 1d) was also observed in the bioaugmentation treatments T3 (non-sterile soil) and T5 (sterile soil), apparently due to the oxidation of these nitrophenol pollutants by PNP monooxygenase (PnpA) from strain SJ98. Nitrite concentrations reached as high as 26.8 ± 3.3 μg g^−1^ dw on day 12 in T3 and 28.4 ± 2.4 μg g^−1^ dw on day 16 in T5; these peak concentrations corresponded to the day when the pollutants were completely removed. The molar ratio of PNP, 3M4NP, and 2C4NP removal to the accumulation of nitrite was approximately 1:0.7 in T5. Moreover, the nitrite concentration increased more rapidly in T3 than in T5 during the first 12 days; this corresponded to the period of nitrophenol degradation. After the removal of nitrophenols, nitrite concentration began to decrease in both treatments T3 and T5. Moreover, the nitrite in the non-sterile soil (T3) decreased more rapidly than in the sterile soil (T5).

### Abundance of inoculated strain SJ98

**Figure 2:**
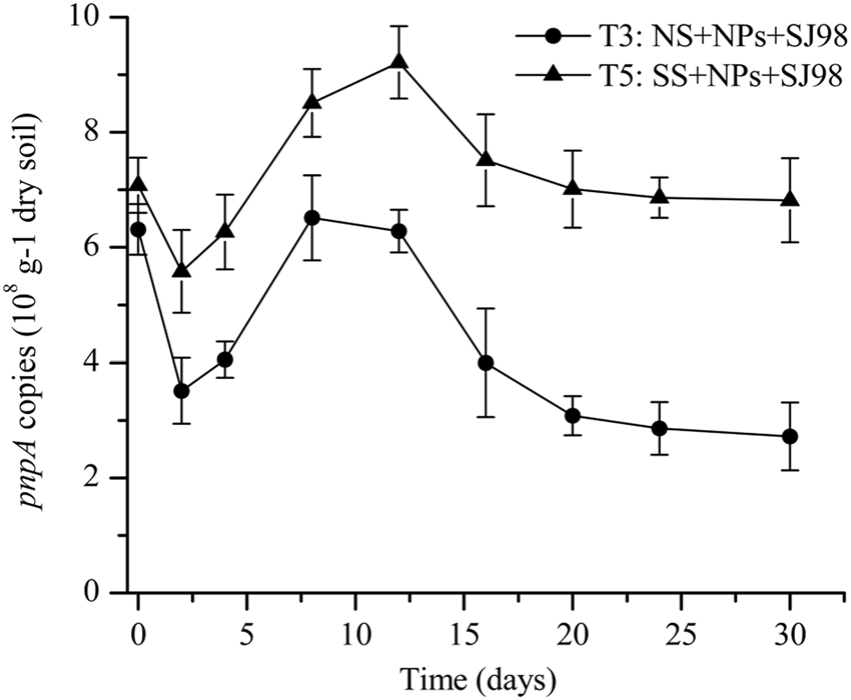
Real-time PCR quantification of *pnpA* in inoculated soil microcosms. Values are means ± standard deviation (n = 3). NS: native soil, SS: sterilized soil, NPs: nitrophenols, and SJ98: *Burkholderia* sp. strain SJ98.

The *pnpA* copy numbers (a surrogate for strain SJ98 abundance) decreased in both bioaugmentation treatments (T3 and T5) during the first 2 days (Fig. 2). Copy numbers subsequently began to increase in a manner consistent with the removal of nitrophenols. The gene copy numbers decreased after the removal of most nitrophenol pollutants (Fig. 2). Moreover, the copy number of *pnpA* on day 42 (2.0 ± 0.47 × 10^8^ g^−1^ dw in T3, and 5.7 ± 0.65 × 10^8^ g^−1^ dw in T5) was slightly, but not significantly, lower than on day 30 (2.7 ± 0.59 × 10^8^ g^−1^ dw in T3, and 6.8 ± 0.73 × 10^8^ g^−1^ dw in T5); this suggests that strain SJ98 survived well even after nitrophenol removal was complete. The abundance of *pnpA* in non-sterile soil (T3) was generally lower than that in sterile soil (T5) throughout the entire incubation period; this is presumably attributable to the resistance and/or competition of the native microbial communities against the strain SJ98 in the non-sterile soil (T3).

### Abundance of indigenous bacteria

**Figure 3:**
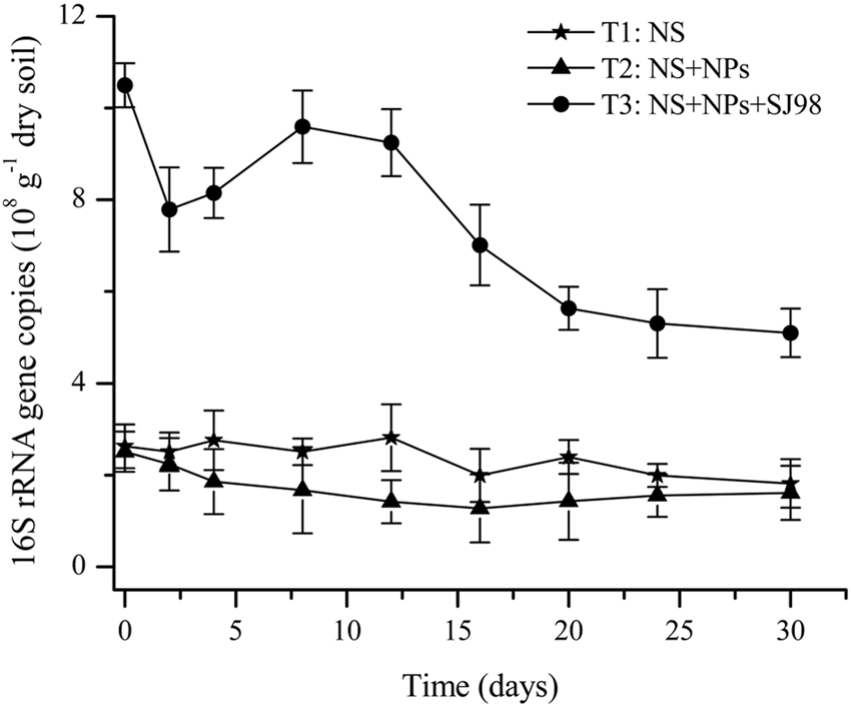
Quantitative changes of total 16S rRNA genes in non-sterile soil microcosms over time by real-time PCR. Values are means ± standard deviation (n = 3). NS: native soil, NPs: nitrophenols, and SJ98: *Burkholderia* sp. strain SJ98.

The total bacteria abundance in non-sterile soil treatments (T1, T2 and T3) was estimated by using real-time PCR targeting the total copy numbers of bacterial 16S rRNA gene (Fig. 3). There was no apparent difference in 16S rRNA gene copy number between the nitrophenol-contaminated treatment (T2) and the uncontaminated microcosm (T1). In the bioaugmentation treatment (T3), the bacterial 16S rRNA gene copy number (5.1 ± 0.53 × 10^8^ to 1.05 ± 0.32 × 10^9^) was significantly higher during the entire incubation period than in T1 (1.8 ± 0.32 × 10^8^ to 2.8 ± 0.29 × 10^8^) and T2 (1.3 ± 0.62 × 10^7^ to 2.5 ± 0.44 × 10^8^); we suspect that this was due to the inoculation of strain SJ98.

### Structure of indigenous bacterial community

Total DNA from 27 soil samples (collected at days 0, 2, 4, 8, 12, 16, 20, 24 and 30 from treatments T1, T2 and T3) was extracted, and high quality DNA was used to amplify the V4 hypervariable region of 16S rRNA gene for HiSeq sequencing. After quality and OTU filtering, a total of 1,414,860 sequences were clustered into 4,343 bacterial OTUs at a 97% similarity threshold. The number of high-quality sequences per sample ranged from 48,583 to 67,505 (Table S2). Good’s coverage values for all of the samples were greater than 98%; this indicates that sequencing captured a majority of the bacterial diversity. The 4,343 OTUs corresponded to 236 genera, 201 families, 172 orders, 107 classes, and 36 phyla. Proteobacteria, Actinobacteria, and Acidobacteria were the most dominant phyla accounting for 42.22%, 37.42%, and 6.12% of abundance, respectively (Fig. S1a). Betaproteobacteria (28.69%), Actinobacteria (25.34%), Thermoleophilia (10.56%), and Alphaproteobacteria (9.07%) were the most dominant classes (Fig. S1b). The most dominant families were Burkholderiaceae (21.32%) and Streptomycetaceae (11.21%, Fig. S1c), and the most dominant genera were *Burkholderia* (21.29%) and *Streptomyces* (11.07%).

### Analysis of bacterial diversity

The α-diversity indexes were used to assess the influence of nitrophenol pollutants on the richness and diversity of indigenous bacteria. We normalized the sequencing reads via resampling, and estimated diversity was calculated from 48,500 sequences per sample (based on the lowest number of reads sampled: 48,583). There was no difference in Sobs, Chao1, or the Shannon indices between the contaminated soil (T2) and the uncontaminated microcosm (T1) during the first 24 days (Table S2). This indicates that the bacterial richness and diversity were not influenced significantly by nitrophenol contamination, in line with the result of real-time PCR analysis. The Chao1 and Shannon indices in the bioaugmentation treatment (T3) were significantly lower than in T1 and T2, which indicate a decline in bacterial richness and diversity following inoculation with strain SJ98. The bacterial richness and diversity in T3 partially recovered over time, but remained lower than in T1 and T2 (Table S2).

### Relationships between environmental variables and bacterial community structure

PCA analysis was performed to visualize the differences in community structures across different microcosms at the OTU level. The first two canonical axes explained 95.43% (axis1: 65.04%, axis2: 30.39%) of variation across 18 samples (Fig. S2). Samples from the contaminated treatment (T2) were distinct from those of the bioaugmented treatment (T3), indicating significant difference in community structure between the two treatments. Within samples from T2, those from the first 16 days were grouped together and distinctly apart from those on day 20, 24, and 30. The nine samples from the bioaugmented treatment (T3) were also divided into several different subgroups; this indicates that incubation had a marked effect on the community structure at the OTU level.

CCA analysis and variation partitioning were performed to determine the correlations between environmental factors and the indigenous bacterial community structure. For the contaminated treatment (T2), 61.53% of the variance was explained by the first two axes (axis1: 49.36%, axis2: 12.17%; Fig. S3a); PNP (*r* ^2^ = 0.9740, *p* = 0.001), 3M4NP (*r* ^2^ = 0.9588, *p* = 0.001), 2C4NP (*r* ^2^ = 0.9691, *p* = 0.001), and nitrite (*r* ^2^ = 0.9006, *p* = 0.002) were determined to have significantly influenced the bacterial community structure. For the bioaugmented treatment (T3), 79.16% of variance was explained by the first two axes (axis1: 72.94%, axis2: 6.22%; Fig. S3b); PNP (*r* ^2^ = 0.8694, *p* = 0.007), 3M4NP (*r* ^2^ = 0.8117, *p* = 0.019 < 0.05), and 2C4NP (*r* ^2^ = 0.6661, *p* = 0.045 < 0.05) were determined to have significantly influenced the bacterial community structure. Meanwhile, nitrite (*r* ^2^ = 0.5281, *p* = 0.138 > 0.05) did not significantly impact the community structure in T3. Strain SJ98 (*r* ^2^ = 0.9722, *p* = 0.001) was shown to be the most important factor that influenced the bacterial community structure during the degradation of nitrophenols.

### Comparison of bacterial community structure at genus level

**Figure 4:**
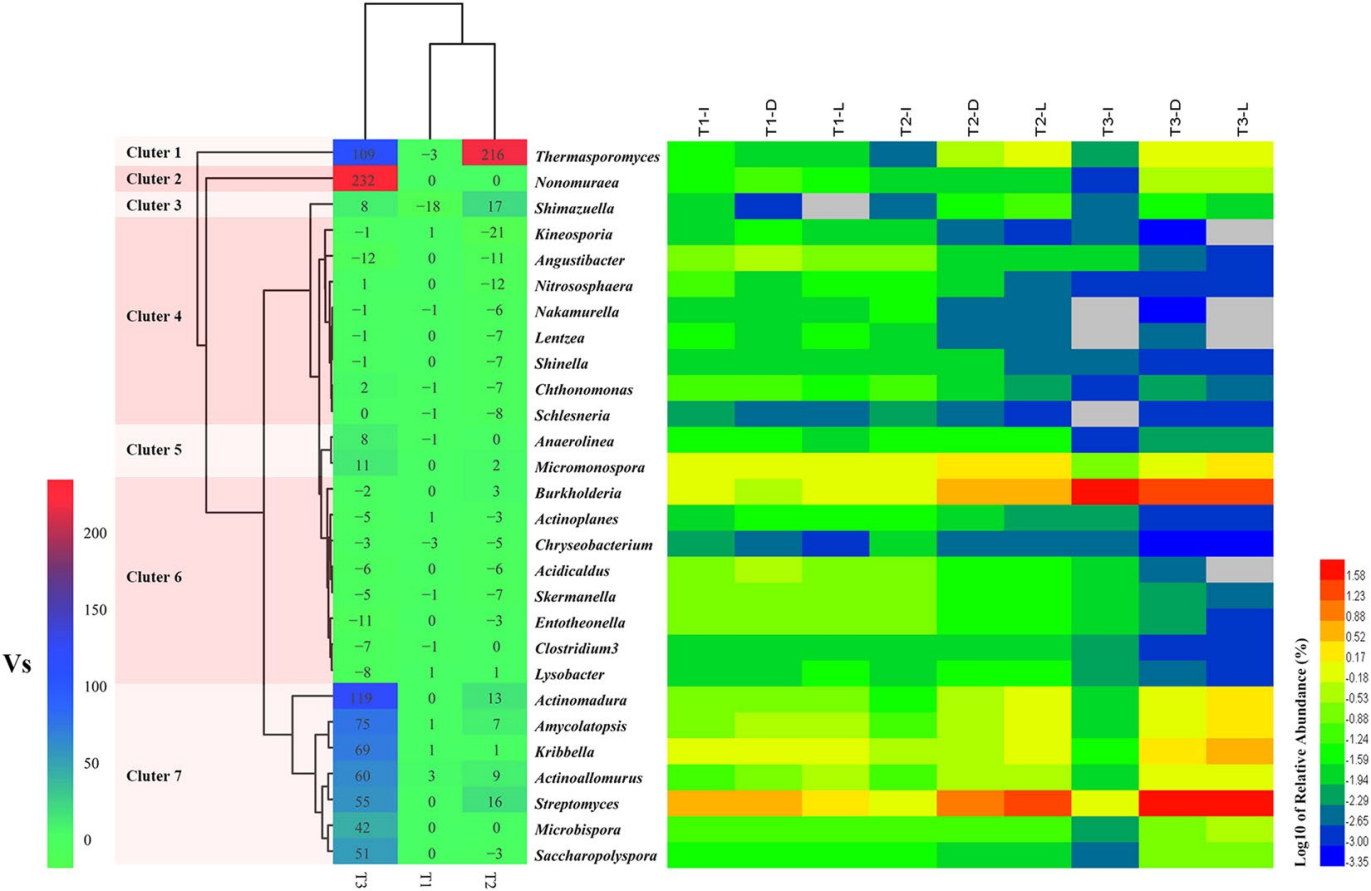
Representative shifts in bacteria genera in uncontaminated (T1), contaminated (T2), and bioaugmented (T3) treatments. The Euclidean-distance dendrogram is based on the shift level across the three treatments and the heatmap profiles showing the shifts level (Left) and relative abundance (Right) of 28 representive genera. The shift level was calculated by: defining the relative abundance of given genus at day 0 and day 30 as R1 and R2, respectively; defining the shift values as Vs with the unit “fold”, where if R1 > R2, Vs = –(R1-R2)/R2 (represents a decrease in relative abundance), and if R1 < R2, Vs = (R2-R1)/R1 (represents an increase in relative abundance). For each of the three samples (T1, T2, and T3), the initial (relative abundance on day 0), mid-degradation (day 8), and late stage (day 30) are shown.

A hierarchically clustered heat-map was generated based on the 27 representative genera whose relative abundance changed more than 5-fold in any treatment after 30 days of incubation. *Burkholderia* was also included because it is the genus of strain SJ98 (Fig. 4). Overall, almost all genera remained stable in the uncontaminated microcosm (T1) except for *Shimazuella*, whose relative abundance decreased 18-fold. In contrast, in the nitrophenol-contaminated soil (T2), the relative abundance of six genera increased and that of 11 genera decreased. In the bioaugmentation treatment (T3), the relative abundance of 12 genera increased and that of 7 genera decreased after 30 days of incubation. An analysis of Euclidean distance showed that T1 and T2 were grouped together and distinctly away from T3. Specifically, the 28 genera were grouped into 7 clusters (Fig. 4).

Cluster 1 is comprised solely of the genus *Thermasporomyces*; the relative abundance of this genus decreased slightly in T1 (3-fold), but increased drastically in T2 (216-fold) and T3 (109-fold) over the 30 day incubation period. *Nonomuraea* remained stable in both T1 and T2, but increased 232-fold in T3. The relative abundance of *Shimazuella* (cluster 3) decreased 18-fold in T1, increased 17-fold in T2, and increased 8-fold in T3. *Kineosporia*, *Nitrososphaera*, and *Schlesneria* (cluster 4) decreased 21-, 12-, and 8-fold, respectively in T2 but remained relatively stable in T3. *Anaerolinea* and *Micromonospora* (cluster 5) increased 8- and 11-fold, respectively, in T3; meanwhile, they both remained stable in T1 and T2. There were two distinctive sub-clusters within cluster 6. One was comprised of *Acidicaldus* and *Skermanella*; the relative abundance of both of these decreased in both T2 and T3. The other sub-cluster was comprised of *Entotheonella*, *Clostridium*, and *Lysobacter*, which maintained relatively stable in T2 but decreased in T3. Seven genera affiliated with cluster 7 increased significantly in T3 (42- to 119-fold increases). Moreover, several genera from this cluster also increased in T2; for example, the relative abundance of *Streptomyces* increased from 1.32% to 23.00% between day 0 and day 30.

## Discussion

Methyl-parathion, fenitrothion, and dicapthon are rapidly degraded in the environment^1–3^; therefore, the environmental contamination is more likely due to the presence of PNP, 3M4NP, and 2C4NP. In recent years, several studies have described the remediation of PNP-contaminated soil^14^ and soils contaminated with PNP as well as other pollutants^22–24^; however, to our knowledge, there are no documented cases of bioremediation of 3M4NP-contaminated soil, or of soils contaminated with mixtures of PNP, 3M4NP, and 2C4NP. In the present study, we describe the bioaugmentation of soil contaminated with PNP, 3M4NP, and 2C4NP by a single microorganism (*Burkholderia* sp. strain SJ98). Strain SJ98 is able to mineralize PNP, 3M4NP, and 2C4NP; therefore, no toxic metabolites are accumulated in the environment.

Successful bioaugmentation depends not only on the catabolic capability of the introduced microorganisms against the target pollutants, but also on survival of the inoculum in a complex environment^15, 21, 25, 26^. We have shown that strain SJ98 played a crucial role in removing PNP, 3M4NP, and 2C4NP from the contaminated soil. The higher degradation rate of 2C4NP compared with PNP and 3M4NP in both bioaugmentation treatments (T3 and T5) is likely due to the higher affinity and catalytic efficiency of PnpA for 2C4NP compared with PNP and 3M4NP^32, 33^. The abundance of strain SJ98 was assessed by quantifying the copy number of the catabolic gene *pnpA* by RT-qPCR, rather than traditional plate counting, which is a time-consuming and labor-intensive method that may underestimate the population^34^. The abundance of strain SJ98 increased in presence of nitrophenols, and subsequently decreased after the complete removal of the pollutants; these results are consistent with other reports of the inoculated microorganisms in numerous bioaugmentation studies^16, 18, 21, 24, 26^. Previous reports have described the opposition to non-indigenous species by indigenous grass^35^ and soil^21, 24^ communities. The lower abundance of strain SJ98 in T3 (non-sterile soil) than in T5 (sterile soil) during the bioaugmentation period suggested that some indigenous microorganisms resisted against and/or competed with strain SJ98 in the soil containing nitrophenols. A report has also linked poor survival of the introduced bacteria with bioaugmentation failure^36^. In this study, we have shown that strain SJ98 was able to utilize local resources and survive well in the soil after PNP, 3M4NP, and 2C4NP were removed and a new community was established.

The indigenous microorganisms in natural environments have shown the potential to mitigate the adverse effects of xenobiotic pollutants^24, 37, 38^. For example, the indigenous microbes in Everglades soils can mineralize 20% PNP^37^ and the microbial communities in sediment-water systems have been reported to degrade up to 60% PNP^38^. It was recently shown that 70% PNP, 80% *meta*-nitrophenol, and 50% *ortho*-nitrophenol mineralization was achieved by indigenous microbes in soils simultaneously contaminated with these nitrophenol isomers^24^. In our study, the higher degradation rate of PNP in the un-inoculated non-sterile soil (T2) compared with the un-inoculated sterile soil (T4) indicated that there are indigenous microorganisms capable of utilizing PNP. Furthermore, the relative abundance of *Streptomyces*, which is able to degrade monocyclic aromatic compounds^39^, as well as indigenous *Thermasporomyces*, *Shimazuella*, *Actinomadura*, and *Actinoallomurus*, increased significantly in the non-sterile contaminated soil (T2) compared to the non-sterile native soil (T1). Therefore, we propose that these genera are capable of degrading PNP, although it is unclear if all of the genera are required. In contrast, the indigenous bacteria are unable degrade 3M4NP and 2C4NP. A possible explanation for the higher degradation rates of 3M4NP and 2C4NP in the bioaugmented non-sterile soil (T3) compared to the bioaugmented sterile soil (T5) is that some indigenous bacteria can take advantage of the intermediate metabolites generated by strain SJ98, thereby accelerating 3M4NP and 2C4NP degradation. To date, numerous pure bacterial cultures have been isolated that are able to utilize PNP as the sole carbon and energy source, and both gram-negative and gram-positive PNP-utilizers have been shown to degrade 3M4NP and 2C4NP using the enzymes involved in PNP catabolism^6, 32, 33^. Interestingly, the indigenous bacteria in our experimental soil appeared to selectively degrade PNP, but not 3M4NP or 2C4NP. Therefore, the isolation of indigenous PNP degraders, which may have different catabolic functions and enzymes than those already documented, is recommended.

Microbial community structure and function can remain relatively stable in the environments during perturbation if there is a high species richness^40, 41^. The effects of contamination and inoculation on the native microbial community should be minimal during bioaugmentation. In our study, contamination did not significantly alter the bacterial species richness and diversity during the first 24 days; this is likely due to activation energy required to alter a complex biological system within a limited incubation period. However, Festa *et al*.^19^ did observe a decline in the microbial richness and diversity of soils contaminated with polycyclic aromatic hydrocarbons compared to uncontaminated soils. We observed a reduction in the microbial richness and diversity following bioaugmentation (T3); this is presumably due to the dominance of strain SJ98, which in turn hampered the detection of rare species. This is supported by the increase in bacterial diversity in T3 that corresponded to the decline in the abundance of strain SJ98, and is in line with the notion that dominance by inoculated strains may cause a significant reduction in microbial complexity during bioaugmentation^24, 27^.

Both contamination and bioaugmentation can impact the bacterial community structure. The higher relative abundances of *Actinomadura*, *Streptomyces*, and *Actinoallomurus* in T3 than in T2 suggested a synergism between strain SJ98 and these indigenous bacteria during pollutant removal. This is in contrast with previous reports showing antagonistic relationships between inoculated strains and indigenous bacteria during the bioremediation of 4-chloronitrobenzene^16^ and polycyclic aromatic hydrocarbons^21^. The relative abundance of *Kineosporia*, *Nitrososphaera*, and *Schlesneria*, among others, decreased in T2 but remained relatively stable in T3. A possible explanation is that pollutants may inhibit the growth of these genera; nevertheless, bioaugmentation likely conferred protection on these taxa. Although inoculation led to a significant increase in the abundances of *Nonomuraea*, *Anaerolinea*, *Micromonospora*, *Kribbella*, *Microbispora*, and *Saccharopolyspora*, we suspect that these groups are not directly related to nitrophenols degradation since their abundances were not increased in T2. It is generally accepted that highly abundant microbes in the environment drive the primary ecological functions of the microbial community^42^. In the present study, inoculation of strain SJ98 increased the abundance of numerous species and protected some indigenous bacteria from the inhibitory effects of contamination, leading to the establishment of a new bacterial community after the complete removal of PNP, 3M4NP, and 2C4NP. The resulting community is likely highly resilient, as the resilience of some species along with an increase in the abundance of other species can maintain community stability during environmental perturbations^24, 43^.

## Conclusions

We demonstrate successful remediation of a PNP-, 3M4NP-, and 2C4NP-contaminated soil via bioaugmentation with *Burkholderia* sp. strain SJ98. Given the pollutant degradation capabilities and survival abilities of strain SJ98, as well as its potentially positive influence on the indigenous bacterial community structure, we conclude that strain SJ98 is an excellent candidate for bioremediation of nitrophenol-contaminated soils. This is the first demonstration of the simultaneous removal of PNP, 3M4NP, and 2C4NP via bioaugmentation, and provides new insights for the bioremediation of nitrophenol-contaminated sites.

## Materials and Methods

### Soil sample collection and characteristics

The soil was collected from a non-contaminated cropland (37° 25′ 54.7″ N, 121° 31′ 13.5″ E) on the campus of the Yantai College of China Agricultural University in Yantai, China. The top layer (0–15 cm) of the soil was collected and sieved through a 2-mm mesh before being stored moist at 4 °C until use. The total organic carbon and nitrogen contents of the soil were 0.86 ± 0.09% and 0.074 ± 0.008%, respectively. The soil had a pH of 7.38 ± 0.14 and a moisture content of 10.64 ± 0.12%.

### Setup of soil microcosm

Microcosms were set up in 250-mL glass bottles containing 110.6 g wet weight (100 g dw) of native soil. Samples in the sterile group were sterilized by autoclaving at 121 °C for 30 min three times every other day. Five different treatments were set up in triplicate: T1 - native soil (NS); T2 - native soil with PNP, 3M4NP, and 2C4NP; T3 - native soil with the three nitrophenols (NPs) and strain SJ98; T4 - sterilized soil (SS) with nitrophenols; and T5 - sterilized soil with nitrophenols and strain SJ98. The microcosms were allowed to equilibrate at 30 °C for 72 h prior to starting the experiment. PNP, 3M4NP, and 2C4NP were then added to the appropriate soil and mixed to give final concentrations of 45 μg g^−1^ dw for each compound (based on preliminary experiments).

Strain SJ98 was grown in lysogeny broth at 30 °C and cells were harvested during the exponential growth phase. The cells were washed twice and resuspended in 0.85% sterile saline before being inoculating at ~0.72 × 10^9^ colony forming units per g dw. An equivalent volume of sterile saline was added to treatments not containing the strain to standardize the final moisture content (~16%) in the microcosms. The bottles were then thoroughly mixed and kept at 30 °C in the dark for the course of the experiment. Samples were taken periodically for analysis.

### Analytical methods

The concentrations of PNP, 3M4NP, and 2C4NP in the soil microcosms were determined by high-performance liquid chromatography. Soil samples (0.5 g) were withdrawn at regular intervals, mixed with 1 mL of methanol, vortexed rigorously for 10 min, and centrifuged at 15,000 × g at 4 °C for 10 min. The supernatant was analyzed on an Agilent 1200 system (Agilent Technologies, Palo Alto, CA, USA) equipped with a diode array detector and an Agilent ZORBAX Eclipse XDB-C18 column (250 mm × 4.6 mm, 5 μm particle size). The analytical specifications have been previously described^33^. PNP, 3M4NP, and 2C4NP had retention times of 8.7, 13.3, and 11.4 min, respectively, and were quantified at 320 nm. Nitrate concentrations were determined spectrophotometrically^44^.

### DNA extraction and real-time quantitative PCR

Total DNA was extracted from 0.5 g of soil from each sample in triplicate using the MoBio UltraClean^TM^ Soil DNA Isolation Kit (MoBio Laboratories, Carlsbad, CA, USA) according to the manufacturer’s instructions. The DNA concentrations and qualities were determined using a NanoDrop 2000 UV-Vis Spectrophotometer (Thermo Scientific, Wilmington, DE, USA). Real-time quantitative PCR of the catabolic gene *pnpA* was performed on a 7500 Fast Real-Time PCR System (Applied Biosystems, Foster City, CA, USA). The 20 μL PCR reaction mixture contained 10 μL of 2 × TransStart Tip Green qPCR SuperMix (TransGen Biotech, Beijing, China), 1 μL of template DNA, 0.4 μL of passive reference dye, and 0.5 μM of each primer (*pnpA*-F and *pnpA*-R, Table 1). Each sample was measured in triplicate. The PCR program was 5 min incubation at 95 °C, followed by 45 cycles of: 30 s at 95 °C, 30 s at 60 °C, and 30 s at 72 °C. To confirm the specificity of the PCR product, a melting curve analysis was performed by continuously measuring fluorescence during a temperature increase from 60 °C to 95 °C. Total bacterial abundance was determined by real-time quantitative PCR of the 16S rRNA gene using the primers BACT1369F/PROK1541R and the probe TM1389F according to previously published methods^45^. Standard curves for real-time PCR assays have been previously described^24^; however, the fragments of the almost complete *pnpA* were amplified with the primers *pnpA*10/*pnpA*1215 (Table 1).

**Table 1:** Primers and probe used in this study.

Primers	Sequence (5′-3′)^*^	Purpose	Reference
*pnpA*-F	CGTCGCAACGAATGTCTTCTATG	Quantifying *pnpA* for real-time PCR	This study
*pnpA*-R	CATACGACGACGCACTTCCTC		This study
*pnpA*10	CTTGAAGGAGTGGTCGTTGTTGG	Amplifying *pnpA* for standard curve	This study
*pnpA*1215	TTACGCTGCAAGCTTAAGAGGC		This study
BACT1369F	CGGTGAATACGTTCYCGG	Quantifying 16S rRNA for real-time PCR	45
PROK1541R	AAGGAGGTGATCCRGCCGCA		45
TM1389F	CTTGTACACACCGCCCGTC		45
F27	AGAGTTTGATCMTGGCTCAG	Amplifying 16S rRNA for standard curve	16
R1492	TACGGYTACCTTGTTACGACTT		16
F515	GTGCCAGCMGCCGCGGTAA	Amplifying 16S rRNA for HiSeq sequencing	51
R806	GGACTACHVGGGTWTCTAAT		51

### HiSeq sequencing and data processing and analysis

The universal primers F515 and R806 (Table 1) were used to amplify the V4 hypervariable region of 16S rRNA gene for high-throughput sequencing (Novogene Bioinformatics Institute, Beijing, China). The sequence data were analyzed using USEARCH version 9.0^46^. Briefly, the paired reads were merged and filtered using the “-fastq_mergepairs” and “-fastq_filter” commands, respectively. Sequences were then clustered into operational taxonomic units (OTUs) using a 97% identity threshold via dereplication (-derep_fulllength), and chimera filtering and clustering (-cluster_otus) were performed. A compiled OTU table was constructed by mapping the reads to OTUs (-usearch_global). Mothur (version 1.36.1) used to classify OTU representative sequences using Silva Release 123 (http://www.mothur.org/) according to the standard operating procedures^47^.

Coverage, observed OTUs (Sobs), richness (Chao1 index), and diversity (Shannon index) were calculated using Mothur and used to estimate the α-diversity of each sample. To analyze the succession of bacterial communities at the genus-level, heat map profiles were generated using heat map package in R^48^. Principal component and canonical correlation analyses (PCA and CCA, respectively) were performed in R using the vegan package^49^, and were used to investigate the relationships between the samples and environmental variables^50^. Monte Carlo permutation tests were applied to the models with 999 unrestricted permutations at a 5% significance level. P < 0.05 was considered to be statistically significant for all statistical analyses.

### Data availability

Raw sequencing data is available at the Sequence Read Archive of the National Center for Biotechnology Information (BioSample accession No. SAMN06006582- SAMN06006608).

## Electronic supplementary material

Supplementary information
