# Supplementary material for: Effect of inoculation of Burkholderia sp. strain SJ98 on bacterial community dynamics and para-nitrophenol, 3-methyl-4-nitrophenol, and 2-chloro-4-nitrophenol degradation in soil
Source: Sci Rep. 2017 Jul 20;7:5983. doi: 10.1038/s41598-017-06436-0 (PMC5519733; doi:10.1038/s41598-017-06436-0)
Supplement: Supplementary file 1 — Supplementary information [file 41598_2017_6436_MOESM1_ESM.pdf]

Effect of inoculation of *Burkholderia* sp. strain SJ98 on bacterial community dynamics and *para*-nitrophenol, 3-methyl-4-nitrophenol, and 2-chloro-4-nitrophenol degradation in soil

Jun Min[1#], Bin Wang[1,2#] & Xiaoke Hu[1*]

[1] Key Laboratory of Coastal Biology and Bioresource Utilization, Yantai Institute of Coastal Zone Research, Chinese Academy of Sciences, Yantai, Shandong, China.

[2] University of Chinese Academy of Sciences, Beijing, China.

[#] These authors contributed equally to this work.

[*]Correspondence and requests for materials should be addressed to X.H. (email: xkhu@yic.ac.cn; tel: 86-535-2109127)

Table S1. The pre-experiment of PNP, 3M4NP and 2C4NP degradation in order to find out the optimal concentration of each pollutant which can be removed completely in a 30-day incubation period.

		nitrophenols concentration (μg g^{-1} ds)					
		PNP		3M4NP		2C4NP	
		0 d	30 d	0 d	30 d	0 d	30 d
Groups with different nitial nitrophenol (μg g^{-1} ds)	10	11.4±0.7	ND	10.8±0.4	ND	10.5±0.6	ND
	20	19.7±1.5	ND	20.8±1.0	ND	19.3±1.2	ND
	30	31.8±1.4	ND	29.7±1.6	ND	31.2±1.7	ND
	50	48.8±2.5	ND	52.2±4.3	ND	47.2±5.4	ND
	70	73.5±4.2	13.5±6.74	71.6±7.4	11.7±4.2	68.8±2.3	5.4±2.7
	100	103.2±5.4	39.4±5.3	98.8±2.1	32.8±3.2	105.2±6.7	23.6±7.4

Strain SJ98 was inoculated at a final concentration of approx. 0.6×10^{9} CFU g^{-1} dw.

[a] ND: Not detected

Table S2. Number of sequences retrieved by the Hiseq sequencing analysis, number of OTUs clustered at 97% similarity level, and coverage, sobs , chao1 and shannon indexes for the different communities.

	Original dataset			Resampled dataset (48500 sequences in per sample)			
	Time (day)	Numbers of sequences	Numbers OTUs	Coverage (%)[a]	sobs[a]	Chao1[a]	Shannon[a]
T1	0	56007	2631	98.36 ±0.03	2513.02 ±10.17	3386.21 ±54.84	6.08±0.023
	2	51054	2510	98.39 ±0.02	2469.33 ±6.44	3294.5 ±34.19	5.99±0.021
	4	51690	2437	98.48 ±0.02	2388.94 ±6.54	3124.82 ±33.1	5.96±0.024
	8	48583	2403	98.47 ±0.00	2401.7 ±1.13	3129.39 ±6.1	5.90±0.022
	12	60916	2596	98.38 ±0.03	2408.73 ±12.42	3243.29 ±60.01	5.92±0.024
	16	48770	2452	98.43 ±0.01	2447.71 ±2.02	3246.35 ±10.89	6.02±0.020
	20	50421	2252	98.41 ±0.02	2221.63 ±5.43	3169.1 ±34.71	5.76±0.022
	24	50495	2417	98.36 ±0.02	2384.44 ±5.62	3231.49 ±30.69	5.91±0.021
	30	55410	2403	98.43 ±0.03	2297.7 ±9.6	3177.97 ±54.94	5.89±0.023
T2	0	55596	2724	98.31 ±0.03	2609.62 ±10.01	3443.2 ±49.19	6.17±0.023
	2	60562	2839	98.34 ±0.03	2653.93 ±11.9	3438.87 ±53.09	6.13±0.024
	4	56136	2730	98.39 ±0.03	2613.39 ±10.09	3374.35 ±44.74	6.13±0.024
	8	58636	2673	98.4 ±0.03	2521.99 ±10.83	3305.53 ±54.63	6.01±0.024
	12	51480	2539	98.37 ±0.02	2491.94 ±6.27	3296.97 ±34.33	5.88±0.026
	16	67505	2789	98.39 ±0.04	2516.11 ±13.94	3280.66 ±62.05	5.91±0.025
	20	50856	2489	98.5 ±0.02	2454.23 ±5.45	3125.36 ±27.87	5.76±0.027
	24	49067	2440	98.28 ±0.01	2430.25 ±3.05	3099.37 ±19.12	5.78±0.027
	30	55659	2203	98.53 ±0.03	2102.53 ±9.51	2825.61 ±44.81	5.03±0.029
T3	0	55403	1092	99.02 ±0.02	1025.76 ±7.82	1731.46 ±52.85	2.51±0.032
	2	55777	1052	99.03 ±0.02	983.2 ±7.88	1666.63 ±56.7	2.46±0.030
	4	63490	1228	98.94 ±0.03	1074.55 ±10.5	1871.37 ±72.9	2.58±0.032
	8	66890	1383	98.87 ±0.03	1184.25 ±11.94	1992.08 ±76.51	2.84±0.033
	12	62621	1318	98.89 ±0.03	1168.43 ±11.08	1947.97 ±73.53	3.09±0.033
	16	60859	1565	98.71 ±0.03	1413.33 ±11.22	2251.51 ±68.74	3.63±0.034
	20	54147	1032	99.03 ±0.02	1278.03 ±7.14	2276.55 ±51.32	3.46±0.030
	24	60455	1221	98.94 ±0.02	1398.81 ±9.96	2171.51 ±66.78	3.73±0.030
	30	48797	1210	99.14 ±0.00	1367.4 ±1.56	2200.91 ±13.52	3.82±0.027

[a]Values based on 48500 random sequences per sample. Resampling and calculating of these indices were executed 10^3 times to obtain the mean values.

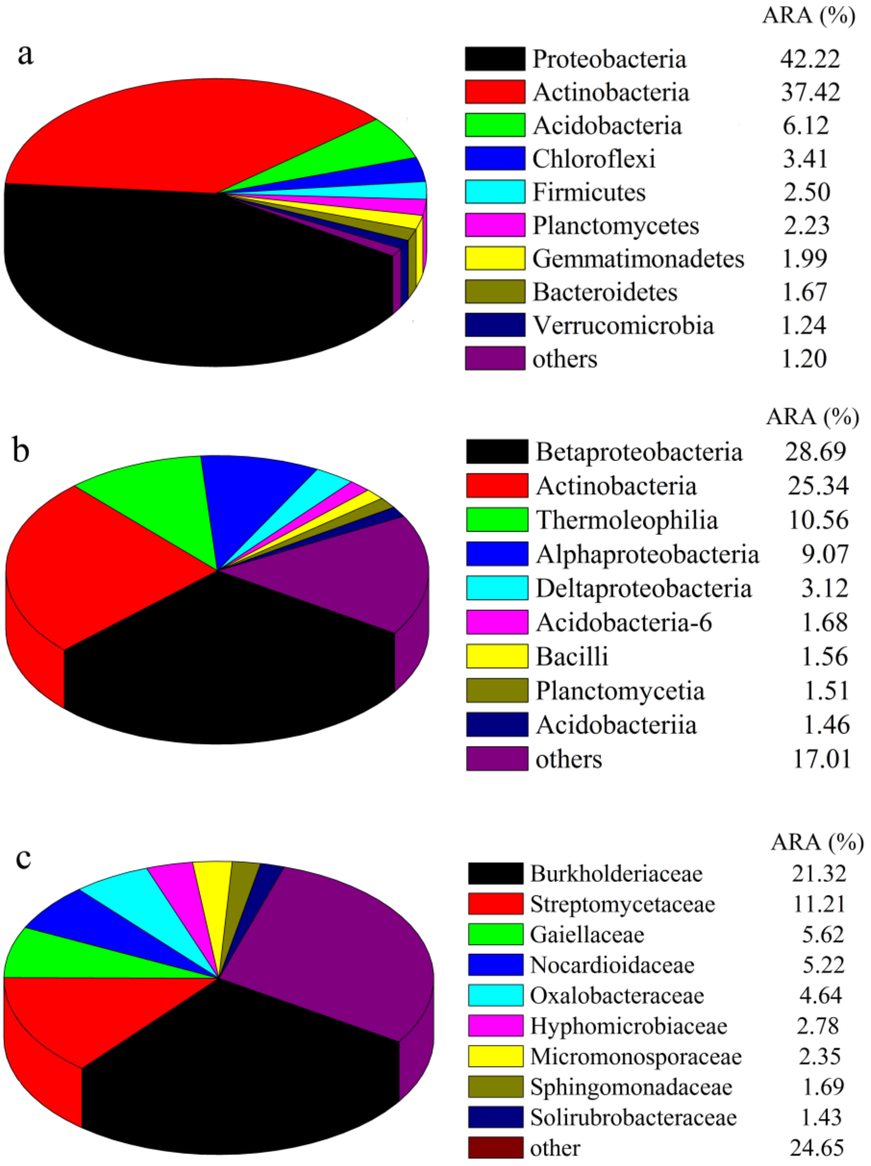

Figure S1. Overall bacterial communities at phyla (A), classes (B) and families (C) in 27 soil samples. ARA: Average relative abundances.

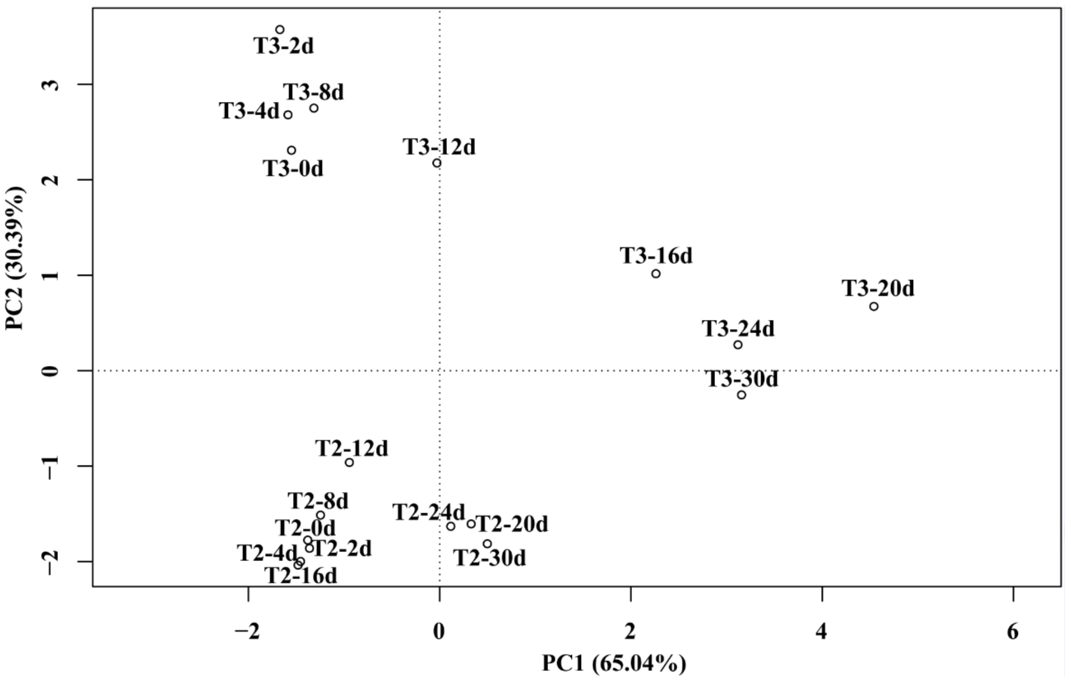

Figure S2. PCA ordination plots of samples of contaminated (T2) and bioaugmented (T3) treatments based on relative abundance of OTUs. Only the first two dimensions are shown. The rings represent samples at different time.

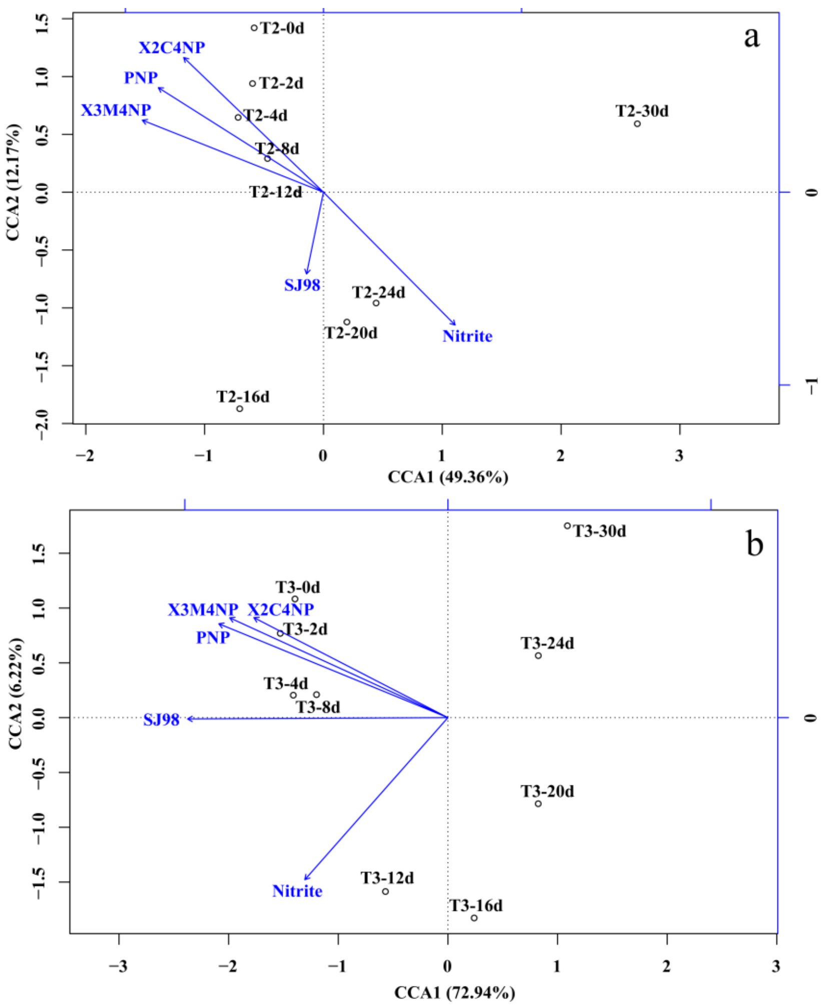

Figure S3. CCA ordination plots showing the relationship between bacterial community structure (A: contaminated treatment T2, B: bioaugmented treatment T3) and environmental variables. Only the first two dimensions are shown. The rings represent samples at different time.
